# Coronary Artery Disease in Patients on Dialysis: Impact of Traditional Risk Factors

**DOI:** 10.3390/medicina60081251

**Published:** 2024-08-01

**Authors:** Dario Nakić, Petra Grbić Pavlović, Marina Vojković, Mira Stipćević, Jogen Patrk, Marin Bištirlić, Zoran Bakotić, Jelena Vučak Lončar, Ivana Gusar

**Affiliations:** 1 Department of Nephrology, General Hospital Zadar, Bože Peričića 5, 23000 Zadar, Croatia; dnakic@unizd.hr (D.N.); petricagrbic@hotmail.com (P.G.P.); marina.vojkovic03@gmail.com (M.V.); 2 Department of Health Studies, University of Zadar, Splitska 1, 23000 Zadar, Croatia; jvucaklon@unizd.hr; 3 Department of Cardiology, General Hospital Zadar, Bože Peričića 5, 23000 Zadar, Croatia; mira.stipcevic@gmail.com (M.S.); jogen.patrk@bolnica-zadar.hr (J.P.); marin.bistirlic@gmail.com (M.B.); zbakotic@gmail.com (Z.B.); 4 Department of Endocrinology, General Hospital Zadar, Bože Peričića 5, 23000 Zadar, Croatia

**Keywords:** coronary artery disease, end-stage kidney disease, risk factors

## Abstract

*Background and Objectives*: End-stage kidney disease (ESKD) is a major risk factor for cardiovascular morbidity and mortality. This study aims to evaluate the contribution of traditional risk factors to the development of coronary artery disease (CAD) in patients on dialysis. *Materials and Methods*: In this study, 54 patients on dialysis with angina symptoms or a positive exercise stress test underwent coronary angiography. Lesions with obstruction >70% lumen diameter of the coronary artery were considered significant. Traditional risk factors included hypertension, diabetes, smoking, dyslipidemia, age, gender, and time spent on dialysis. *Results*: Out of 54 participants, 41 (75.92%) were men and 13 (24.07%) women. CAD was present in 34 (62.96%) patients, and 20 (37.03%) patients were without CAD. The average age of the participants was 66.51 years. In the group with CAD, the average age was 69.52 years with an average time spent on dialysis of 2.73 years. In the group without CAD, the average age was 61.40 years with a time spent on dialysis of 2.35 years. Hypertension was present in 92.59% of all participants and 97.05% of those with CAD. Diabetes was present in 41.17 patients with CAD and 40% of those without CAD. Dyslipidemia was present in 76.47 participants with CAD and in 40% of those without CAD. Smoking was noticed in 35.29% of the participants with CAD and 57.14% of those without CAD. Besides hypertension, significant predictors for the development of CAD in patients on dialysis were dyslipidemia (OR 3.698, Cl 1.005–13.608, *p* = 0.049) and age (OR 1.056, Cl 1.004–1.110, *p* = 0.033). *Conclusions*: Among the traditional risk factors, hypertension, dyslipidemia, and age are the predictors for the development of CAD in patients on dialysis. Further large randomized clinical studies are needed to clarify the role of traditional risk factors for CAD in patients with ESKD.

## 1. Introduction

Cardiovascular disease (CVD) is the leading cause of morbidity and mortality among patients with end-stage kidney disease (ESKD) treated with the different modalities of dialysis. One of the most important manifestations of CVD in chronic kidney disease (CKD) is coronary artery disease (CAD). The prevalence of CAD and the number of significant coronary stenoses increases with the decrease in kidney function [1]. CVD results from traditional risk factors like hypertension, diabetes, dyslipidemia, smoking, and age, but also from non-traditional risk factors like arteriosclerosis, vascular calcifications, endothelial dysfunction, and low-grade inflammation [2,3]. Besides CAD, this process leads to left ventricular hypertrophy (LVH), diffuse fibrosis, left ventricular dilatation, and heart failure [4]. Cardiovascular mortality among patients on dialysis is 10–20 times higher compared to that of the general population of the same age and it is responsible for 50% of deaths among patients with ESKD [5]. According to the results from the US Kidney Data System, the prevalence of CAD in the CKD population is 39% versus 16% in the non-CKD population. The situation among patients undergoing hemodialysis is even worse, with the prevalence of CAD at 42% and that of CVD at 70% [6].

ESKD modifies the clinical presentation of the traditional symptoms of CAD. Acute myocardial infarction (AMI) with chest pain was presented in 44% of patients on dialysis compared with 68% of no-dialysis patients [7]. The recognition of CAD is more difficult with the persistence of anemia and fatigue common among patients on dialysis. Low functional capacity and achieving the threshold for angina pain also limit recognition of symptoms of angina pain in these patients [8]. Electrocardiogram (ECG) changes in angina pain are less common among patients on dialysis. A lower proportion of patients on dialysis presented with ST-segment changes than non-dialyzed patients. ECG changes are often present in patients with ESKD in the form of left ventricular hypertrophy (LVH), ST-segment, and T-wave abnormalities [9]. Serum biomarkers like cardiac troponin (high-sensitivity troponin-I and troponin-T) have limited sensitivity because they are often persistently elevated in patients with ESKD due to LVH, diastolic dysfunction, and volume overload in the absence of acute coronary syndrome [10].

Coronary angiography (CA) is considered the gold standard for diagnosing coronary stenosis in CAD. Coronary computed tomography angiography (CCTA) and coronary artery calcium score (CACS) might be used with caution in patients with ESKD, but the evidence comparing the accuracy of CCTA with CA in this population is limited. Low specificity could be explained by the high calcium burden in coronary arteries without significant stenosis [11,12]. Considering the limited number of studies on the development of coronary disease in dialysis patients and the presence of well-known risk factors, this study aimed to assess the presence and impact of traditional risk factors of coronary artery disease in dialysis patients. Traditional risk factors for CAD (hypertension, diabetes, dyslipidemia, smoking, and age) as well as time spent on dialysis were analyzed.

## 2. Materials and Methods

### 2.1. Study Design and Participants 

This retrospective observational study was conducted for the period from 2019 to 2023. The patients included in this study were 54 adult patients over 18 years of age receiving dialysis: 51 on hemodialysis and 3 on peritoneal dialysis, all undergoing CA at a single center. 

### 2.2. Data Collection

The age and gender of patients, as well as conventional cardiovascular risk factors (hypertension, diabetes, dyslipidemia, smoking, and history of peripheral vascular disease) and duration of dialysis, were collected from the patients’ medical records.

### 2.3. Procedures

Inclusion indications for coronary angiography (CA) included angina symptoms or a positive exercise stress test (ergometry) in patients undergoing screening for kidney transplantation. Patients who did not have angina symptoms or had a negative exercise stress test (ergometry) were excluded from the study. Significant coronary artery lesions were considered significant if the stenosis was more than 70% of the luminal diameter.

CA was performed via the radial artery; only in cases where access via the radial artery was not possible was the femoral artery used. Lesions were coded according to the degree of obstruction, from normal to non-obstructive (<70% stenosis) and obstructive disease (>70% stenosis). 

### 2.4. Statistical Analysis 

Descriptive statistics for categorical variables are presented as proportions and percentages, while numerical data are presented with the mean and standard deviation. The ANOVA test was used to examine differences in the incidence of coronary artery disease concerning years of dialysis. Logistic regression was performed to identify predictors of coronary artery disease in dialysis patients. A significance level of α = 0.05 was used as a criterion for the statistical significance of the results obtained. The data were analyzed using STATISTICA software version 14.0.0.15 (TIBCO Software Inc., Palo Alto, CA, USA, 2018).

## 3. Results

Of the 54 participants in the study, 41 (75.92%) were men and 13 (24.07%) were women. The most common underlying condition for ESKD was hypertensive/ischemic nephropathy (33%), diabetic nephropathy (28%), glomerulonephritis (22%), polycystic kidney disease (4%), and other conditions (13%). The average age of the participants was 66.51 years (SD = 13.12) (Table 1).

Participants who were diagnosed with coronary artery disease were older than participants who were not diagnosed with coronary artery disease (*p* = 0.026). The difference between participants regarding the duration of dialysis was not recorded (*p* = 0.526) (Table 1).

**Figure 1:**
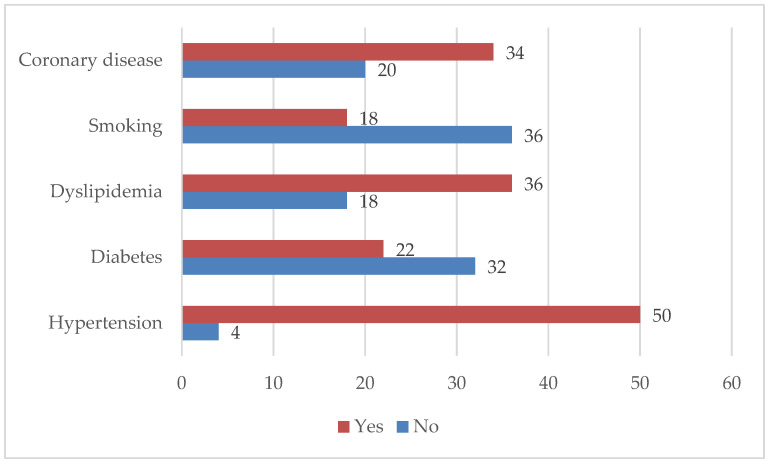
Characteristics of the participants concerning the presence of risk factors (*n* = 54).

Most of the participants had a diagnosis of hypertension (*n* = 50; 92.59%), followed by dyslipidemia (*n* = 36; 66.66%). A total of 18 (33.33%) participants consumed tobacco products. More than half of the participants (32.96%) were found to have coronary artery disease on diagnostic coronary angiography (Figure 1).

The presence of hypertension was recorded in almost all participants diagnosed with coronary artery disease (*n* = 33), while a total of 26 patients with coronary artery disease were found to have dyslipidemia. The majority of participants diagnosed with coronary artery disease were men (*n* = 26) and most participants (*n* = 22) did not consume tobacco products (Table 2).

The results of the ANOVA test did not record a statistically significant difference (*p* = 0.120) in the incidence of coronary disease among the participants regarding the duration of dialysis. Therefore, the duration of dialysis, as well as the presence of hypertension due to its presence in almost all subjects with coronary artery disease, was excluded from further analysis.

After applying logistic regression analysis, age (OR 1.056, 95% Cl 1.004–1.110, *p* = 0.033) and dyslipidemia (OR 3.698, 95% Cl 1.005–13.608, *p* = 0.049) were recorded as statistically significant predictors of coronary artery disease in the participants of this study. With an increase of one year of life, the chance of coronary disease increased 1.05 times. Furthermore, participants diagnosed with dyslipidemia had a 3.69 times higher risk of developing coronary artery disease (Table 3).

## 4. Discussion

Patients with ESRD who require dialysis have a significantly increased risk of morbidity and mortality from CVD. CAD is the major clinical manifestation of a worse prognosis for these patients. It is more likely to develop acute myocardial infarction (AMI) than stable angina as an initial clinical manifestation of CAD. AMI is more likely to be a non-ST-segment elevation myocardial infarction (nSTEMI) than an ST-segment elevation myocardial infarction (STEMI) [13,14]. Relatively common in dialysis patients is a sudden death perhaps because of changes in electrolytes, volume, and drug concentration which may trigger arrhythmias in patients with LVH and heart failure, which are common in these patients [4]. 

Hypertension is the most common CVD risk factor in patients with ESKD. In our study group, it was present in 92% of all participants and in 97% of those with CAD, which confirms hypertension as the most important risk factor for CAD not only in patients with ESKD. Similar results were found in the study by Vasuveda et al. in patients with stable CAD and ESKD who underwent coronary artery bypass grafting (CABG) with a hypertension prevalence of 97% [15]. In a study on predictors of CAD progression in high-risk patients with recurrent symptoms, Fatah et al. reported a 71% prevalence of hypertension [16].

In addition, this study found that dyslipidemia is an important risk factor for the development of CAD in ESKD patients, increasing the risk by 3.69 times. The treatment of dyslipidemia in patients with ESKD is controversial. The benefit of statin-based treatment in reducing serious vascular events is lower with the decline in the glomerular filtration rate, with no clear evidence for dialysis patients [17].

Also, age was found to be a risk factor for the development of CAD in patients on dialysis with a chance of developing CAD of 1.05 per year. This was not noticed for the years spent on dialysis. Chen et al. found, in a study of Asian dialysis patients, that higher all-cause mortality was associated with a dialysis time greater than 3 years in patients undergoing CABG [18]. In this study, both groups, with and without CAD, spent less than 3 years on dialysis; those with CAD spent 2.73 years and those without CAD spent 2.35 years on dialysis, which may suggest that a much longer duration of dialysis may be associated with a higher risk of developing CAD.

Diabetes mellitus (DM) and chronic kidney disease (CKD) are among the strongest risk factors for cardiovascular disease (CVD) [19]. This includes every aspect of CVD, including atherosclerotic CVD (ASCVD), heart failure (HF), valvular heart disease, peripheral arterial disease (PAD), stroke, and arrhythmias [20]. In addition, the overall mortality associated with CVD in these conditions remains high. The coexistence of these conditions has additive and sometimes multiplicative effects on these outcomes [21]. Based on the results of this study, 41.17% of patients with CAD were diabetic patients, but 40% without CAD had diabetes as an additional risk factor, so we found no association between diabetes and the risk of CAD in patients on dialysis.

According to the literature, smoking increases all causes of death in dialysis patients and leads to a 10-year reduction in life expectancy. It also leads to a 36% higher risk of cardiovascular events [22,23]. It is important to emphasize that several studies have found that smoking increases the incidence of peripheral vascular events and heart failure. However, an increased incidence of cerebrovascular or coronary vascular events was not found [24]. Furthermore, smoking accelerates atherosclerotic disease, impairs endothelial function, increases blood pressure, and increases sympathetic outflow [23]. Smoking before dialysis causes acute elevations in blood pressure, leading to more antihypertensive medications being prescribed which interfere with adequate fluid withdrawal due to intradialytic hypotension [23]. In this study, 35% of patients were active smokers, which is a significantly higher percentage than the results found in the literature (15%) [22], but is very similar to the results of 31% of smokers from reports of previous Croatian epidemiological studies in the general population [25]. An association between smoking and the progression of coronary artery disease in smokers among the dialysis population was not found in this study.

There are different treatment options for CAD in advanced CKD: conservative and interventional strategies such as percutaneous coronary intervention (PCI) and coronary artery bypass grafting (CABG) [26]. Reports from the ISCHEMIA-CKD trial provided no evidence that the initial invasive strategy reduced the risk of death or non-fatal myocardial infarction in these patients compared with the initial conservative strategy [27]. For the invasive approach, there is still no clear evidence of the benefits of CABG or PCI. In the SYNTAX trial [28], there is an advantage of CABG, EXCEL showed no significant difference between PCI and CABG [29], and in a national study from Taiwan, PCI with a drug-eluting stent was associated with better survival in dialysis patients [30].

Among patients on dialysis, there is a high prevalence of asymptomatic patients, estimated to be between 42 and 54% according to some reports [31]. The KDIGO guidelines for cardiovascular disease in dialysis patients recommend annual CAD evaluation in patients with diabetes on the waiting list for transplantation as a non-invasive screening [32]. Currently, routine invasive screening for CAD is not part of clinical practice for these patients. However, in the future, it may be necessary to consider implementing this option to prevent CAD and promptly improve dialysis patients’ treatment. Although this study provides valuable results and information that may potentially contribute to deciding on the introduction of additional preventive diagnostic procedures for dialysis patients, the fact that this is a relatively small sample of patients from a single healthcare institution that cares for the population of a relatively limited geographical area should also be taken into account. Furthermore, we did not collect other parameters of prognostic value in this study, such as laboratory measurements and pharmacotherapy, which may also have a significant impact on patient outcomes.

## 5. Conclusions

Hypertension, dyslipidemia, and age were significant predictors for the development of CAD in patients on dialysis, and better control of hypertension and dyslipidemia is necessary to prevent the occurrence of CAD in these patients. Further large randomized clinical trials are needed to clarify the role of traditional risk factors for CAD in patients with ESKD.

## Figures and Tables

**Table 1:** Age of participants and duration of dialysis (*n* = 54).

Variable	Overall (*n* = 54)				Coronary Artery Disease (*n* = 34)				No Coronary Artery Disease (*n* = 20)				*p*
	Mean	Min	Max	SD	Mean	Min	Max	SD	Mean	Min	Max	SD	
Age *	66.51	29	90	13.12	69.52	38	90	10.93	61.40	29	89	15.13	0.026
Dialysis length *	2.59	1	11	2.12	2.73	1	11	2.15	2.35	1	7	2.10	0.526

Min—Minimum; Max—Maximum; * presented in years.

**Table 2:** Distribution and characteristics of participants with regard to the presence of coronary artery disease (*n* = 54).

Variable	Coronary Artery Disease (*n* = 54)				Total	
	Yes (*n* = 34)		No (*n* = 20)			
Man	Number	Percentage	Number	Percentage	Number	Percentage
	26	76.47	15	75.00	41	75.92
Woman	8	23.52	5	25.00	13	24.07
Hypertension	33	97.05	1	5.00	34	62.96
Diabetes	14	41.17	8	40.00	22	40.74
Dyslipidemia	26	76.47	8	40.00	34	62.96
Smoking	12	35.29	22	57.14	34	62.96

**Table 3:** Logistic regression analysis for predictors of coronary artery disease in dialysis patients (*n* = 54).

Variable	B	S.E.	Wald	df	*p* *	OR	95% CL	
							Lower	Upper
Age	0.055	0.026	4.547	1	0.033	1.056	1.004	1.110
Diabetes	−0.003	0.654	0.000	1	0.997	0.997	0.277	3.595
Dyslipidemia	1.308	0.665	3.872	1	0.049	3.698	1.005	13.608
Smoking	0.526	0.680	0.598	1	0.439	1.692	0.447	6.409
Constant	−4.084	1.914	4.553	1	0.033	0.017		

* Logistic regression analysis.

## Data Availability

The authors will make available the data supporting this article’s conclusions on request.
